# Parathyroid Hormone Administration Improves Bone Marrow Microenvironment and Partially Rescues Haematopoietic Defects in *Bmi1*-Null Mice

**DOI:** 10.1371/journal.pone.0093864

**Published:** 2014-04-04

**Authors:** Ruinan Lu, Qian Wang, Yongli Han, Jianyong Li, Xiang-Jiao Yang, Dengshun Miao

**Affiliations:** 1 The State Key Laboratory of Reproductive Medicine,The Research Center for Bone and Stem Cells, Department of Human Anatomy, Nanjing Medical University, Nanjing, China; 2 Department of Hematology, The First Affiliated Hospital of Nanjing Medical University, Nanjing, China; 3 The Rosalind & Morris Goodman Cancer Research Center and Department of Biochemistry, McGill University, Montreal, Quebec, Canada; 4 Department of Medicine, McGill University Health Center, Montreal, Quebec, Canada; The University of Hong Kong, Hong Kong

## Abstract

The epigenetic regulator Bmi1 is key in haematopoietic stem cells, and its inactivation leads to defects in haematopoiesis. Parathyroid hormone (PTH), an important modulator of bone homeostasis, also regulates haematopoiesis, so we asked whether PTH administration improves bone marrow microenvironment and rescues the haematopoietic defects in *Bmi1*-null mice. The mice were treated with PTH1-34 (containing the first 34 residues of mature PTH), an anabolic drug currently used for treating osteoporosis, and compared with the vehicle-treated *Bmi1* ^-/-^ and wild-type littermates in terms of skeletal and haematopoietic phenotypes. We found that the administration significantly increased all parameters related to osteoblastic bone formation and significantly reduced the adipocyte number and PPARγ expression. The bone marrow cellularity, numbers of haematopoietic progenitors and stem cells in the femur, and numbers of lymphocytes and other white blood cells in the peripheral blood all increased significantly when compared to vehicle-treated *Bmi1^-/-^* mice. Moreover, the number of Jagged1-positive cells and percentage of Notch intracellular domain-positive bone marrow cells and protein expression levels of Jagged1 and NICD in bone tissue were also increased in *Bmi1* ^-/-^ mice upon PTH1-34 administration,whereas the up-regulation of PTH on both Notch1 and Jagged1 gene expression was blocked by the Notch inhibitor DAPT administration. These results thus indicate that PTH administration activates the notch pathway and partially rescues haematopoietic defects in *Bmi1*-null mice, further suggesting that haematopoietic defects in the animals are not only a result of reduced self-renewal of haematopoietic stem cells but also due to impaired bone marrow microenvironment.

## Introduction

The haematopoietic system is highly complex, extremely active and very important in health and diseases. Two key components of the system are the haematopoietic stem cells (HSCs) and their bone marrow microenvironment. HSCs exist in a relatively quiescent state and are the source of all the differentiated blood cells. They execute long-term self-renewal and multi-lineage differentiation functions [1], [2]. The bone marrow microenvironment is referred as the HSC niche, a microenvironment where different cell types and extracellular matrix molecules dictate stem cell self-renewal and progeny production [3]. It has been appreciated that bone marrow mesenchymal stem cells (BM-MSCs) provide a structural scaffold for haematopoiesis [4]. Besides reticular fibroblasts, macrophages, adipocytes and endothelial cells, osteoblasts are also part of the stromal cell support system in the bone marrow. Osteoblastic cells regulate the HSC niche [5]. HSC differentiation occurs in direct proximity to osteoblasts within the bone marrow cavity. HSCs reside in medullar niches, mainly in the endosteum where osteoblasts and stromal cells provide HSCs with signals for maintaining the homeostatic quiescent state [5], [6].

The polycomb protein Bmi1 (B lymphoma Mo-MLV insertion 1) was identified originally as an oncogenic partner of c-Myc in murine lymphomagenesis. Bmi1 is required for self-renewal and postnatal maintenance of HSCs [7] and also important for neural stem cells from the central and peripheral nervous systems [8], [9]. Mice lacking *Bmi1* display defects in haematopoiesis and development of the central and peripheral nervous systems [10]. It has been reported that progressively impaired haematopoiesis in the bone marrow of *Bmi1* ^-/-^ mice results in decreased cell numbers and replacement of large areas of haematopoiesis in the bone marrow by adipocytes [10]. Our previous studies indicate that Bmi1 maintains self-renewal of BM-MSCs by inhibiting the expression of p27, p16, and p19 and alters the cell fate of BM-MSCs by enhancing osteoblast differentiation and inhibiting adipocyte differentiation at least in part by stimulating expression of the lsyine deacetylase Sirt1 [11]. These results indicate that Bmi1 deficiency results in both defects in haematopoiesis and osteoblastic bone formation; however, it is unclear whether defects in haematopoiesis caused by Bmi1 deficiency are associated with impaired bone marrow microenvironment for haematopoiesis.

Parathyroid hormone (PTH) is a peptide comprised of 84 amino acids and is the main regulator of calcium and phosphate homeostasis. It is secreted from the cells of the parathyroid glands and acts through a class B G-protein–coupled receptor, PTH receptor (PTHR). In fact, the N-terminal 34 amino acids of mature PTH are sufficient for activation of signaling through PTHR [12]. PTH1-34, a recombinant human parathyroid hormone analog containing the 34 residues, is currently used as an anabolic drug to treat osteoporosis [13]. PTH increases osteoblast production rate and inhibits apoptosis of osteoblasts, thereby leading to a rapid increase in skeletal mass as well as improvement of bone micro-architecture and strength [14]. Our previous study demonstrated that PTH1-34 administration significantly increased cortical and trabecular bone mass with augmented osteoblast number and activity [15]. Furthermore, available evidence also suggests that PTH stimulates haematopoiesis [5], [16]–[18]. Osteoblasts in transgenic mice expressing a constitutively active form of PTHR only in the osteoblast lineage support accumulation of twice more HSCs than normal [5]. PTH not only exerts anabolic action by stimulating osteoblastic bone formation, but also upregulates haematopoiesis by improving bone marrow microenvironment. These findings raise the important question whether PTH administration is able to rescue haematopoietic defects from *Bmi1* deficiency though improving the bone marrow microenvironment. To answer this question, *Bmi1* ^-/-^ mice were treated with PTH1-34 and compared with vehicle-treated *Bmi1* ^-/-^ and wild-type mice. The results indicate the administration improves the microenvironment and rescues haematopoietic defects in *Bmi1*-null mice, thereby revealing a potential value of PTH1-34, an anabolic drug for osteoporosis, for repairing haematopoietic defects.

## Materials and Methods

### Mice and genotyping

*Bmi1* ^-/-^ mice (129Ola/FVB/N hybrid background), kindly provided by Professor Anton Berns, The Netherlands Cancer Institute, [10] had been backcrossed 10 to 12 times on the C57BL/6J background. Genotypes of the mutant mice were determined by PCR analysis as described previously [8]. The 1-week-old wild type and *Bmi1* ^-/-^ mice received daily injections of vehicle or of PTH1-34 (80 μg/kg) subcutaneously for 3 weeks. For DAPT (N-[N-(3,5-difluorophenacetyl)-l-alanyl]-S-phenylglycine t-butyl ester) blocking assay, the 4-week-old *Bmi1* ^-/-^ mice received daily injections of PTH1-34 (80 μg/kg) subcutaneously or injections of PTH1-34 plus injections of DAPT (10 μmol/kg) intraperitoneally for 2 days, the same age wild type and *Bmi1* ^-/-^ mice received daily injections of vehicle as controls. All animal experiments were carried out in compliance with and approval by the Institutional Animal Care and Use Committee of Nanjing Medical University (Approval ID 2008-00318).

### Skeletal radiography

Femurs were removed and dissected free of soft tissue. Contact radiographs were taken using a Faxitron Model 805 radiographic inspection system (Faxitron Contact, Faxitron, Germany; 22 kV and 4-minute exposure time). X-Omat TL film (Eastman Kodak Co., Rochester, NY, USA) was used and processed routinely.

### Micro-computed tomography (µCT)

Femurs obtained from 2-week-old mice were dissected free of soft tissue, fixed overnight in 70% ethanol, and analyzed by µCT with a SkyScan 1072 scanner and associated analysis software (SkyScan, Antwerp, Belgium) as described previously [19]. Briefly, image acquisition was performed at 100 kV and 98mA with a 0.9-degree rotation between frames. During scanning, the samples were enclosed in tightly fitting plastic wrap to prevent movement and dehydration. Thresholding was applied to the images to segment the bone from the background. 2D images were used to generate 3D renderings using the 3D Creator software supplied with the instrument. The resolution of the µCT images is 18.2 μm.

### Histology

Tibiae were removed and fixed in PLP fixative (2% paraformaldehyde containing 0.075M lysine and 0.01M sodium periodate) overnight at 4°C and processed histologically as described previously [20]. Proximal ends of tibiae were decalcified in EDTA glycerol solution for 5 to 7 days at 4°C. Decalcified tibiae were dehydrated and embedded in paraffin, after which 5 μm sections were cut on a rotary microtome. The sections were stained with hematoxylin and eosin (H&E) or histochemically for total collagen [21] or alkaline phosphatase activity (ALP) [22], or immunohistochemically as described below.

### Immunohistochemical staining

Osterix, type I collagen, osteopontin, PTHR, Jagged1 and Notch1 were determined by immunohistochemistry as described previously [20], [23]. Polyclonal rabbit Anti-osterix (Abcam, USA), polyclonal goat anti-type I collagen (Santa Cruz, USA), rabbit anti-mouse osteopontin (Millipore, USA), monoclonal anti-PTHR (Millipore, USA), rabbit anti-mouse Jagged1 (Santa Cruz, USA) and rabbit anti-mouse activated Notch1 polyclonal antibody (Unconjugated, Abcam, USA) were employed.

### Quantitative real-time PCR

RNA was isolated from mouse long bones using Trizol reagent (Invitrogen, Inc., Carlsbad, CA, USA) according to the manufacturer's protocol. Reverse-transcription reactions were performed using the SuperScript First-Strand Synthesis System (Invitrogen), as described previously [24]. Real-time PCR was performed using a LightCycler system (Roche, Indianapolis, IN, USA) as described previously [25]. The conditions were 2 μL of LightCycler DNA master SYBR Green I (Roche), 0.25 μM of each 50 and 30 primer and 2 μL of sample and/or H_2_O to a final volume of 20 μL. The MgCl_2_ concentration was adjusted to 3 mM. Samples were amplified for 35 cycles with a temperature transition rate of 20°C/s for all three steps, which were denaturation at 95°C for 10 seconds, annealing for 5 seconds and extension at 72°C for 20 seconds. SYBR green fluorescence was measured to determine the amount of double-stranded DNA. To discriminate specific from nonspecific cDNA products, a melting curve was obtained at the end of each run. Products were denatured at 95°C for 3 seconds, and then the temperature was decreased to 58°C for 15 seconds and raised slowly from 58 to 95°C using a temperature transition rate of 0.1°C/s. To determine the number of copies of target DNA in the samples, purified PCR fragments of known concentration were serially diluted and served as external standards for each experiment. Glyceraldehyde-3-phosphate dehydrogenase (*Gapdh*) was used as the internal control for each reaction. The relative amount of mRNA was normalized to *Gapdh* mRNA. The primer sequences used for the real-time PCR were as described [25], [26].

### Western blot analysis

Proteins were extracted from long bones or cells and quantitated by a kit (Bio-Rad, Mississauga, Ontario, Canada). Protein samples of 30 μg were fractionated by SDS-PAGE and transferred to nitrocellulose membranes. Immunoblotting was carried out as described previously [25] using antibodies against Runx2 (MBL International, Woburn, MA), peroxisome proliferator-activated receptor γ (Ppar-γ, E-8, Santa Cruz, CA, USA), PTHR (clone 3D1.1, Millipore), insulin-like growth factor 1 (IGF-1, clone Sm1.2, Millipore), Jagged1 (Santa Cruz, USA), activated Notch1 (Abcam, USA) and β-tubulin (Santa Cruz, CA, USA). Bands were visualized using enhanced chemiluminescence (ECL, Amersham) and quantitated by Scion Image Beta 4.02 (Scion Corporation, Bethesda, MD, USA).

### Complete blood count (CBC)

Each mouse was bled by retro-orbital puncture for blood cell counts. Blood (20 μl) was collected and mixed with 180 μL Cell-Dyn buffer immediately. Complete blood count was analyzed with a Cell Dyn 3700 counter (Abbott Laboratories, Ill, USA). Two blood samples of each mouse were collected for CBC analysis. The numbers of neutrophils and platelets from all animals were averaged, and the data are presented as means ± standard deviations.

### Flow cytometry

For analysis of HSCs, BM cells were stained with PE-conjugated anti-Sca1 (BioLegend), PE-Cy5.5-conjugated anti-c-Kit (eBioscience), and Alexa Fluor 488-conjugated Mouse Lineage Mixture Antibodies (Invitrogen). The HSCs were defined as Sca-1^+^c-Kit^+^Lin^-^ and the HPCs as Sca-1^+^c-Kit^+^ Lin^+^. All analyses were performed on a FACSCalibur (BD Biosciences).

### Computer-assisted image analysis

After HE staining or histochemical or immunohistochemical staining of sections from six mice of each genotype, images of fields were photographed with a Sony digital camera. Images of micrographs from single sections were digitally recorded using a rectangular template, and recordings were processed and analyzed using Northern Eclipse image analysis software as described previously [23], [27].

### Statistical analysis

Data from image analysis are presented as mean ± s.e.m. Statistical comparisons were performed by use of a two-way ANOVA, with *P*<0.05 considered to be significant.

## Results

### Effect of PTH1-34 on the length of long bones and trabecular bone volume in *Bmi1^-/-^* mice

**Figure 1:**
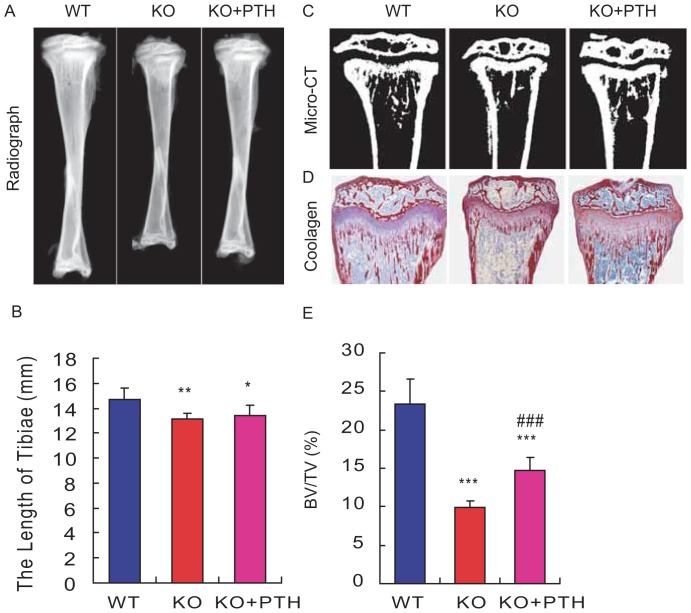
Effect of PTH1-34 on the length of long bones and trabecular bone volume in *Bmi-1^-/-^* mice. Representative radiographs, (B) quantitation of the length of tibiae, (C) 3-dimensional reconstructed longitudinal sections of micro-CT scanning images and (D) micrographs of paraffin sections of the tibiae stained with Siries Red for total collagen from 4-week-old vehicle-treated wild-type (WT) and *Bmi-1^-/-^* mice (KO) and PTH1-34-treated *Bmi-1^-/-^* mice (KO+PTH), magnification, ×50. (E) Quantitation of trabecular bone volume relative to tissue volume (BV/TV, %) in metaphyseal regions. For each genotype, n = 6; *: *p*<0.05, **: *p*<0.01, ***: *p*<0.001, compared to vehicle-treated WT mice; ###: *p*<0.001 compared to vehicle-treated *Bmi-1^-/-^* mice.

To determine whether skeletal growth retardation and osteopenic phenotype caused by Bmi1 deficiency were improved by PTH administration, we treated 1 week old *Bmi1* ^-/-^ and wild-type mice subcutaneously with vehicle or PTH1-34 at 80 μg/kg per day for 3 weeks. Long bones from vehicle-treated wild-type and *Bmi1* ^-/-^ mice and PTH1-34-treated *Bmi1* ^-/-^ mice were analyzed at 4 weeks of ages by radiography and μCT. The lengths of tibiae were shorter in vehicle-treated *Bmi1^-/-^* mice than in their wild-type littermates (Figs. 1A–B). Radiolucency was greater in *Bmi1^-/-^* mice relative to wild-type mice (Fig. 1A). From 3D reconstructed longitudinal sections of the proximal ends of tibiae, it was clear that epiphyses were smaller and trabecular bone volumes were lower in *Bmi1^-/-^* mice than the wild-type mice (Fig. 1C). The length of tibiae was not increased, whereas the trabecular bone volume increased significantly in *Bmi1^-/-^* mice by PTH1-34 administration, but had not reached the normal levels as vehicle-treated wild-type mice (Figs. 1A–C). Consistent with μCT analysis, histological analysis demonstrated that trabecular bone volume was reduced significantly at 4 weeks of age in vehicle-treated *Bmi1^-/-^* mice when compared with their wild-type littermates (Figs. 1D–E). The volume increased significantly in *Bmi1^-/-^* mice upon PTH1-34 administration, but had not reached normal levels as vehicle-treated wild-type mice (Figs. 1D–E). These results demonstrated that osteoporotic phenotypes caused Bmi1 deficiency was reversed partially by PTH1-34 administration.

### Effect of PTH1-34 on osteoblast bone formation in *Bmi1^-/-^* mice

**Figure 2:**
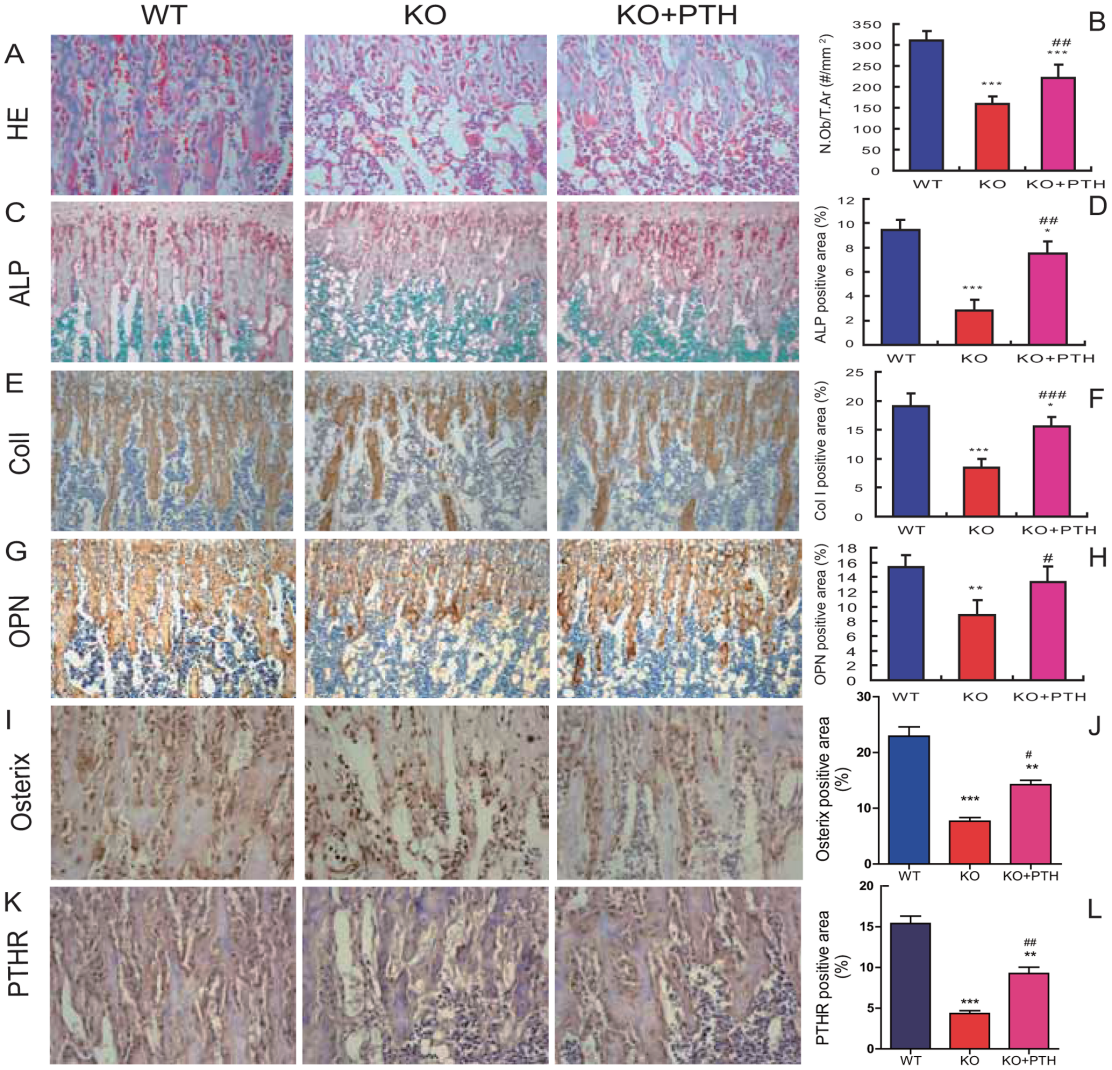
Effect of PTH1-34 on osteoblastic bone formation in *Bmi-1^-/-^* mice. Representative micrographs of paraffin-embedded sections for tibial metaphyseal regions from 4-week old vehicle-treated wild-type (WT) and *Bmi-1^-/-^* mice (KO) and PTH1-34 treated *Bmi-1^-/-^* mice (KO+PTH) stained (A) histologically with hematoxylin & eosin (HE, ×400), (C) histochemically for alkaline phosphatase (ALP, ×200), immunohistochemically for (E) type I collagen (ColI, ×200), (G) osteopontin (OPN, ×200), (I) osterix (x400) and (K) PTHR (x400). (B) Osteoblast counts (#/mm^2^), (D) ALP-positive areas, (F) type I collagen- or (H) OPN- or (J) osterix- or (L) PTHR-immunopositive areas were measured by computer-assisted image analysis. For each genotype, N = 6; *: *p*<0.05, **: *p*<0.01, ***: *p*<0.001, compared to vehicle-treated WT mice; #: *p*<0.05, ##: *p*<0.01, ###: *p*<0.001 compared to vehicle-treated *Bmi-1^-/-^* mice.

**Figure 3:**
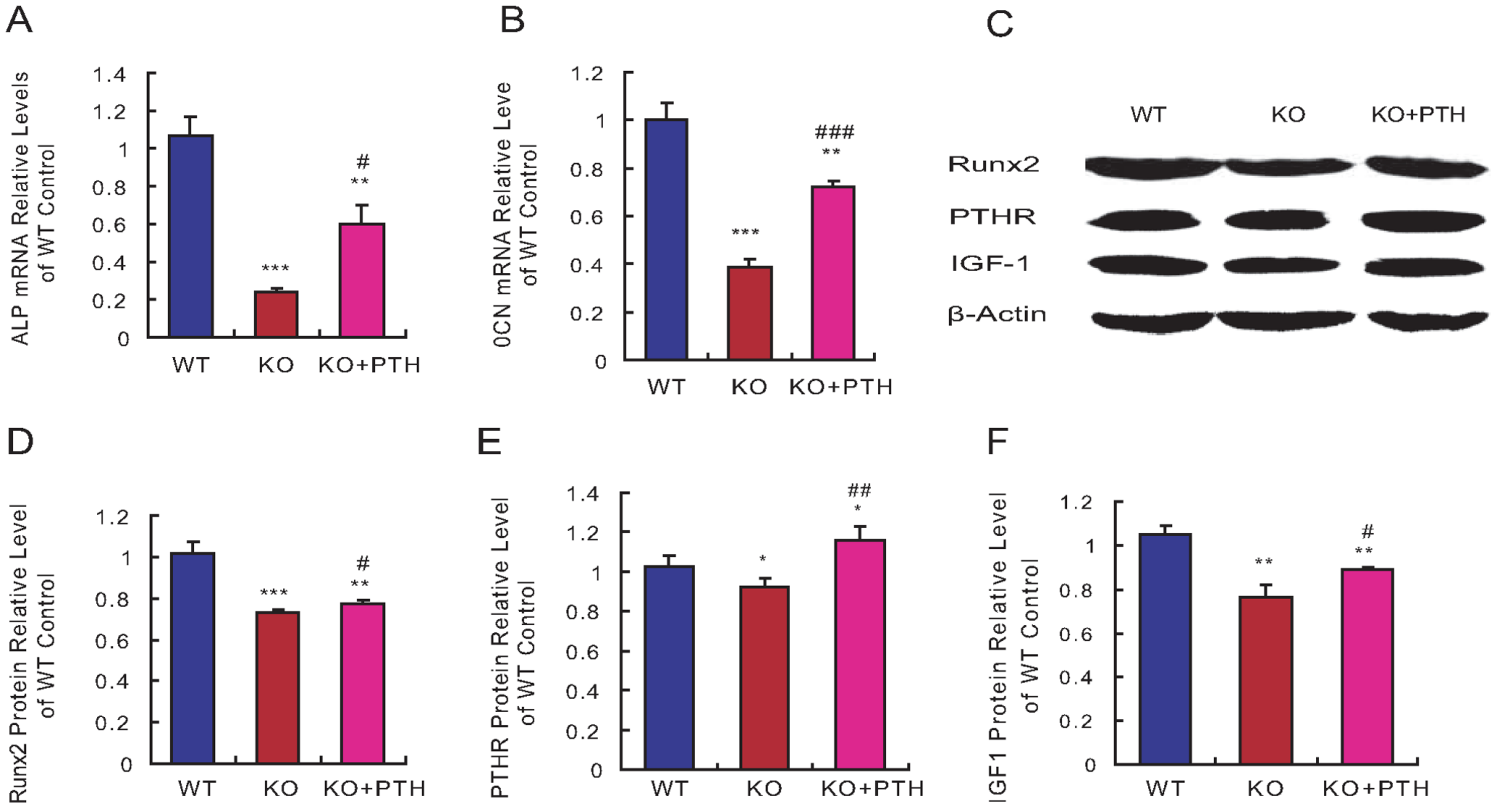
Effect of PTH1-34 on expression of markers for osteoblastic bone formation in *Bmi-1^-/-^* mice. (A–B) Real-time RT–PCR was performed on humerus extracts from 4-week-old vehicle-treated wild-type (WT) and *Bmi-1^-/-^* mice (KO) and PTH1-34 treated *Bmi-1^-/-^* mice (KO+PTH) for determining the expression of (A) alkaline phosphatase (ALP) and (B) osteocalcin. The expression is calculated as a ratio to the GAPDH mRNA level and shown relative to the levels in vehicle-treated WT mice. (C) Western blots of femur extracts from 4-week-old vehicle-treated WT and *Bmi-1^-/-^* mice and PTH1-34-treated *Bmi-1^-/-^* mice for expression of Runx2, PTHR and IGF-1. β-actin was used as loading control for Western blots. (D-F) Runx2, PTHR and IGF-1 protein levels relative to the β-actin level were assessed by densitometric analysis and presented relative to the levels in vehicle-treated WT mice. For each genotype, n = 6; *: *p*<0.05, **: *p*<0.01, ***: *p*<0.001, compared to vehicle-treated WT mice; #: *p*<0.05, ##: *p*<0.01, ###: *p*<0.001 compared to vehicle-treated *Bmi-1^-/-^* mice.

To determine whether the increased trabecular bone volume in *Bmi1^-/-^* mice by PTH1-34 administration was associated with the improvement of osteoblastic bone formation, the number of osteoblasts, ALP activity in osteoblasts, deposition of type I collagen and osteopontin in the bone matrix and expression of osterix and PTHR in osteoblasts were examined by HE staining, histochemical staining for ALP and immunostaining for type I collagen, osteopontin, osterix and PTHR. At 4 weeks of age, the osteoblast number (Figs. 2A and B), ALP-positive areas (Figs. 2C and D), type I collagen (Figs. 2E and F), osteopontin (Figs. 2G and H), osterix (Figs. 2I and J) and PTHR (Figs. 2K and L) were reduced significantly in vehicle-treated *Bmi1^-/-^* mice compared with their wild-type littermates. These parameters all improved significantly in *Bmi1^-/-^* mice upon PTH1-34 administration, but did not reach the levels comparable to their wild-type littermates (Fig. 2). It was noted that PTHR was localized in osteoblasts and stromal cells in bone marrow, but not in bone marrow haematopoietic cells (Fig. 2K). We also examined expression of genes and proteins important for bone formation. RNA and proteins were isolated from long bones for real-time RT-PCR and Western blots. The results showed that the transcript levels of ALP and osteocalcin and the protein levels of Runx2, PTHR and IGF1 were all down-regulated significantly in *Bmi1^-/-^* mice compared with their wild-type littermates (Figs. 3A–F). Importantly, PTH1-34 administration upregulated the levels of the transcripts and proteins in *Bmi1^-/-^* mice although not to the normal levels (Figs. 3A–F), indicating that the defects in osteoblastic bone formation caused by Bmi1 deficiency were partially repaired by PTH1-34 administration.

### Effect of PTH1-34 on bone marrow haematopoietic cells and adipocytes in *Bmi1^-/-^* mice

**Figure 4:**
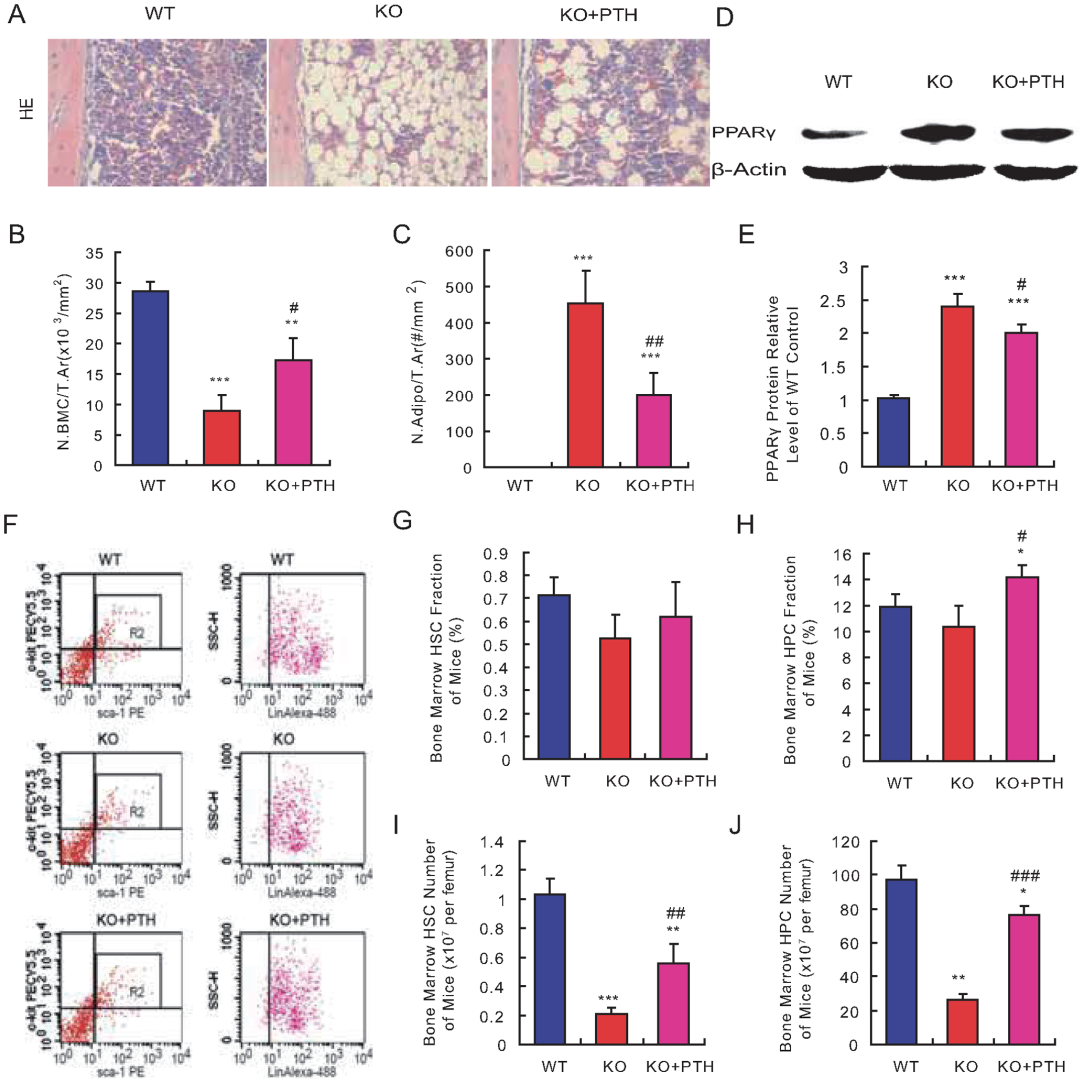
Effect of PTH1-34 on the bone marrow cellularity in *Bmi-1^-/-^* mice. (A) Representative micrographs of paraffin-embedded sections of tibial diaphyseal regions from 4-week-old vehicle-treated wild-type (WT) and *Bmi-1^-/-^* mice (KO) and PTH1-34 treated *Bmi-1^-/-^* mice (KO+PTH) stained with hematoxylin and eosin (HE, ×400). (B) The one marrow cell number and (C) adipocyte number relative to tissue area were measured by computer-assisted image analysis. (D) Western blots of femur extracts from 4-week-old vehicle-treated WT and *Bmi-1^-/-^* mice and PTH1-34-treated *Bmi-1^-/-^* mice for determination of PPARγ expression. β-actin was used as the loading control. (E) The PPARγ level relative to the β-actin level was assessed by densitometric analysis and shown relative to the levels in the vehicle-treated WT mice. (F) Representative graphs of flow cytometry analysis for hematopoietic stem cells (HSCs) and hematopoietic progenitor cells (HPCs) in the bone marrows from 4-week-old vehicle-treated wild-type (WT) and *Bmi-1^-/-^* mice (KO) and PTH1-34- treated *Bmi-1^-/-^* mice (KO+PTH). (G-H) Fractions of Sca-1^+^c-kit^+^Lin^−^ HSCs and Sca-1^+^c-kit^+^Lin^+^ HPCs in the bone marrows. (I–J) The numbers of Sca-1^+^c-kit^+^Lin^-^ HSCs and Sca-1^+^c-kit^+^Lin^+^ HPCs in each femur were assessed and presented relative to the levels in the vehicle-treated WT mice. For each genotype, n = 6; *: *p*<0.05, **: *p*<0.01, ***: *p*<0.001, compared to vehicle-treated WT mice; #: *p*<0.05, ##: *p*<0.01, ###: *p*<0.001 compared to vehicle-treated *Bmi-1^-/-^* mice.

To assess directly whether haematopoietic defects caused by Bmi1 deficiency could be improved by PTH administration, the numbers of bone marrow haematopoietic cells and adipocytes were counted on HE-stained sections of diaphyseal regions in long bones. At 4 weeks of age, the number of bone marrow haematopoietic cells decreased significantly, while the number of bone marrow adipocytes increased dramatically in 4-week old *Bmi-1^-/-^* mice when compared with their wild-type littermates (Figs. 4A–C). When PTH1-34-treated *Bmi-1^-/-^* mice were compared with the vehicle-treated counterparts, the number of bone marrow haematopoietic cells increased significantly, while the number of bone marrow adipocytes was reduced significantly (Figs. 4A–C). We also assessed whether the alterations of bone marrow adipocytes were associated with PPARγ expression changes. Proteins were isolated from long bones and Western blots were performed. Results showed that the expression level of PPARγ was upregulated significantly in *Bmi1* ^-/-^ mice compared to their wild-type littermates (Figs. 4D and E). PTH1-34 administration significantly downregulated PPARγ expression in *Bmi1^-/-^* mice (Figs. 4D–E). To determine whether alterations of bone marrow haematopoietic cells in *Bmi1^-/-^* mice by PTH administration were associated with effects on the HSC and HPC populations, the fraction and numbers of HSCs and HPCs in the bone marrow were analyzed in 4-week old mice by fluorescence-activated flow cytometry. Results showed that the fractions of HSCs (Sca-1^+^c-Kit^+^Lin^-^) and HPCs (Sca-1^+^c-Kit^+^Lin^+^) were reduced insignificantly (Figs. 4F–H), however, total numbers in the femur bone marrow were reduced significantly in *Bmi1^-/-^* mice when compared to their wild-type littermates (Figs. 4I–J). The fractions of HSCs and HPCs, as well as the total numbers of both HSCs and HPCs, increased significantly or remarkably in the femur bone marrow of PTH1-34-treated *Bmi1^-/-^* mice (Figs. 4F–J). These results indicated that PTH administration reverses the low haematopoietic cell number (including HSCs and HPCs) and the high adipocyte count in the bone marrow due to Bmi1 deficiency.

### Effect of PTH1-34 on the peripheral blood cellularity in *Bmi1^-/-^* mice

**Figure 5:**
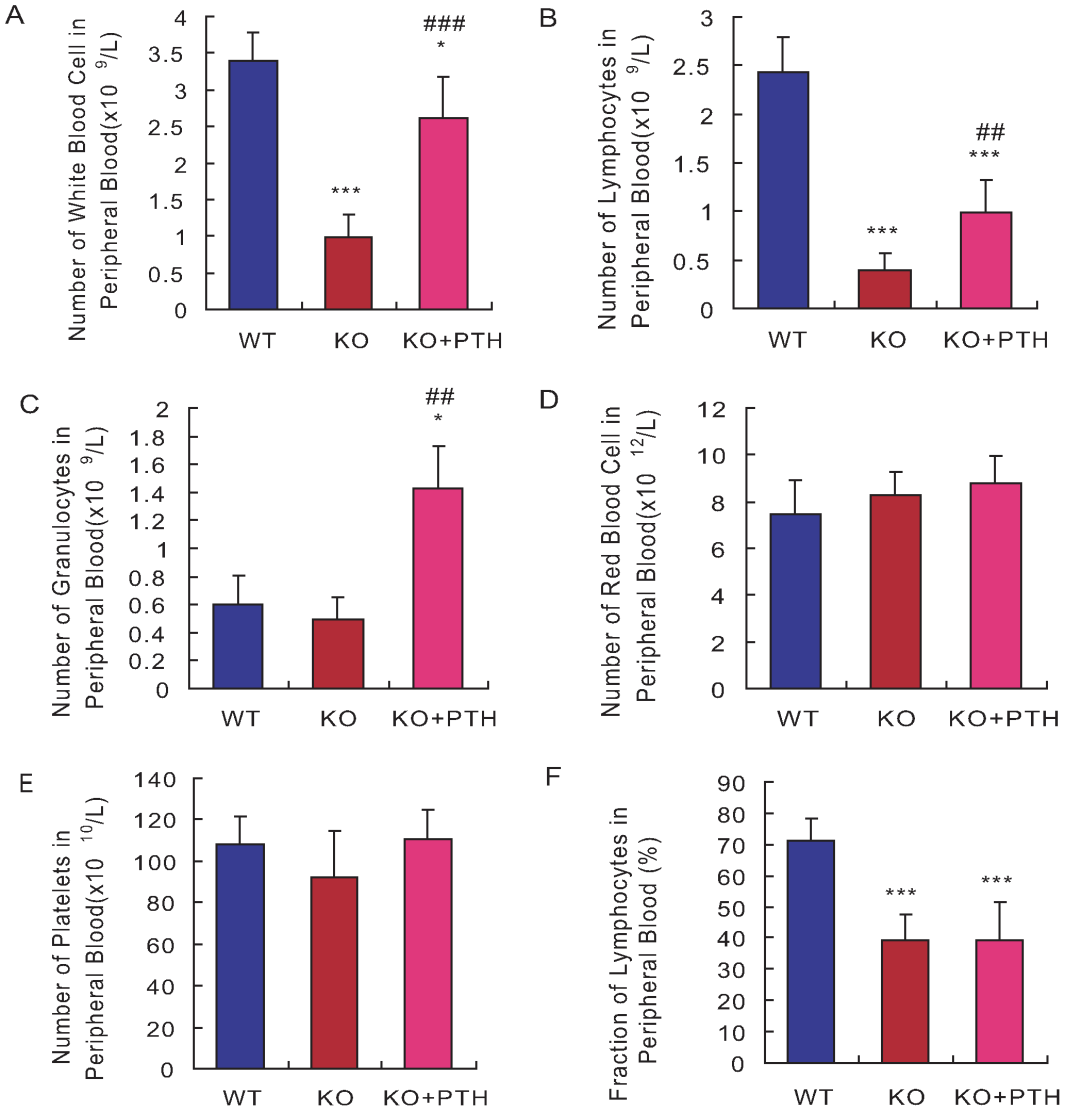
Effect of PTH1-34 on the peripheral blood cellularity in *Bmi-1^-/-^* mice. The peripheral blood from 4-week-old vehicle-treated wild-type (WT) and *Bmi-1^-/-^* mice (KO) and PTH1-34 treated *Bmi-1^-/-^* mice (KO+PTH) were analyzed by a hematological analyzer, to determine the number of white blood cells (A), lymphocytes (B), granulocytes (C), red blood cells (D), platelets (E) and the fraction of lymphocytes (F). For each genotype, n = 6; *: *p*<0.05, ***: *p*<0.001, compared to vehicle-treated WT mice; ##: *p*<0.01, ###: *p*<0.001 compared to vehicle-treated *Bmi-1^-/-^* mice.

To assess whether Bmi1 deficiency resulted in abnormalities of peripheral blood cells and PTH administration could rescue their possible abnormalities, we analyzed peripheral blood from 4-week old vehicle-treated wild-type and *Bmi1^-/-^* mice and PTH1-34-treated *Bmi1^-/-^* mice by use of a hematological analyzer. Specifically, the number of white blood cells, lymphocytes, granulocytes, red blood cells, platelets and the fraction of lymphocytes were evaluated. At 4 weeks of age, the number of white blood cells and lymphocytes in peripheral blood decreased significantly in vehicle-treated *Bmi-1^-/-^* mice when compared to the wild-type littermates (Fig. 5A–B). PTH1-34 administration partially reversed the deficits in *Bmi-1^-/-^* mice, but not to the normal levels as wild-type mice (Figs. 5A–B). The numbers of granulocytes in peripheral blood was slightly reduced insignificantly in vehicle-treated *Bmi-1^-/-^* mice when compared to the wild-type littermates (Fig. 5C) and increased dramatically in PTH1-34-treated *Bmi-1^-/-^* mice (Fig. 5C). The numbers of red blood cells and platelets in peripheral blood were not altered significantly in both vehicle-treated and PTH1-34-treated *Bmi1^-/-^* mice (Figs. 5D–E). The fraction of lymphocytes decreased significantly in both vehicle-treated and PTH1-34-treated *Bmi1^-/-^* mice (Fig. 5F). These results demonstrated that the numbers of white blood cells and lymphocytes and the fraction of lymphocytes decreased significantly in *Bmi1*-deficient mice and that PTH administration increased the numbers of these cells significantly but had minimal impact on the fraction of lymphocytes.

### Effect of PTH1-34 on Notch signaling molecules in *Bmi1^-/-^* mice

**Figure 6:**
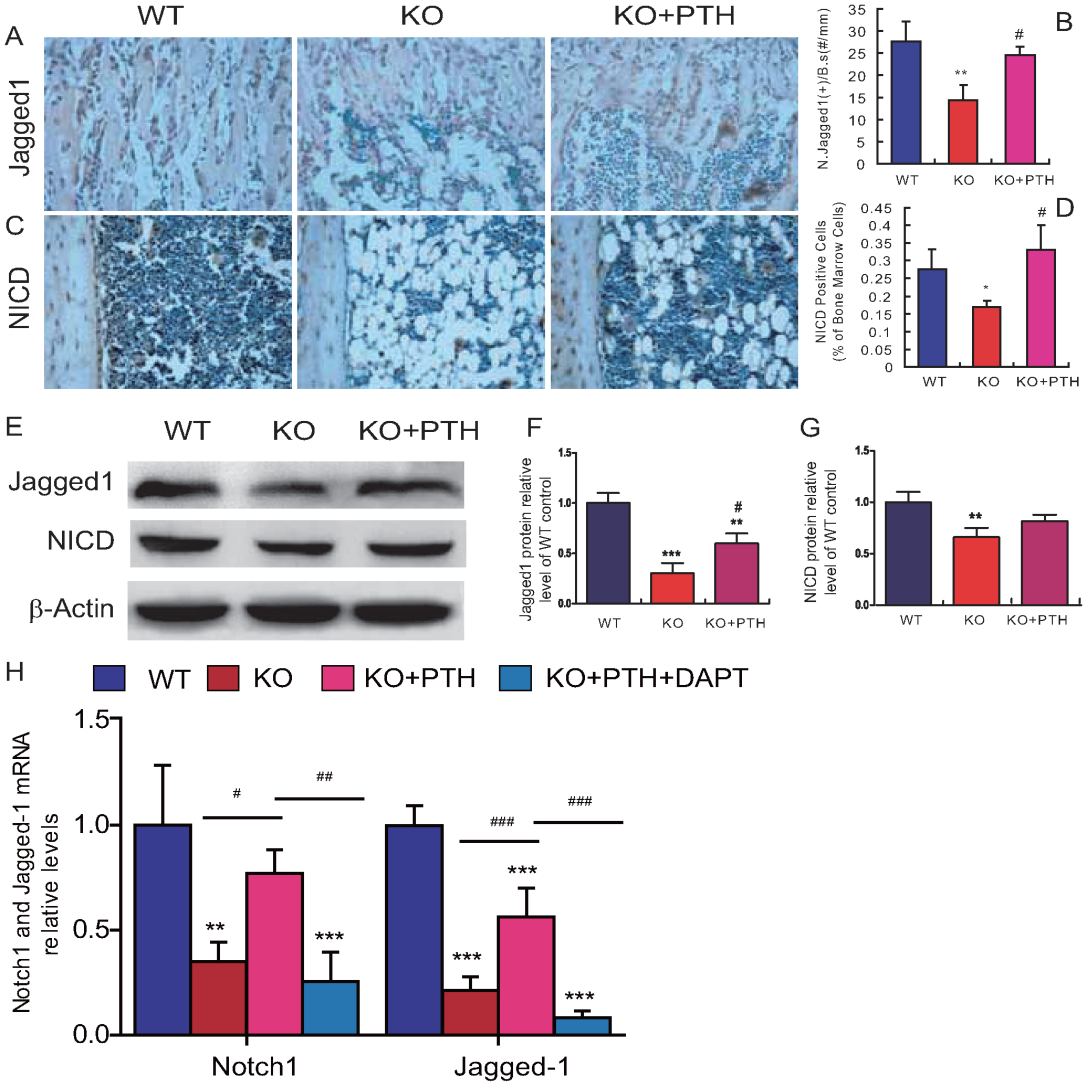
Effect of PTH1-34 on Notch signal pathway-related molecules in *Bmi-1^-/-^* mice. (A–B) Representative micrographs of paraffin-embedded sections of tibiae from 4-week-old vehicle-treated wild-type (WT) and *Bmi-1^-/-^* mice (KO) and PTH1-34- treated *Bmi-1^-/-^* mice (KO+PTH) stained immunohistochemically for the Notch ligand Jagged1 (A) and Notch intracellular domain (NICD, B), magnification, ×400. (C) The number of Jagged1-positive cells relative to bone surface (#/mm^2^) and (D) the percentage of NICD-positive bone marrow cells were measured by computer-assisted image analysis. (E) Western blots were performed on the long bone extracts for expression of jagged1and NICD. β-actin was used as loading control for Western blots. (F) jagged1 and (G) NICD protein levels relative to the β-actin level were assessed by densitometric analysis and presented relative to the levels in vehicle-treated WT mice. (H) Real-time RT–PCR was performed on long bone extracts from vehicle-treated wild-type (WT) and *Bmi-1^-/-^* mice (KO), PTH-treated *Bmi-1^-/-^* mice (KO+PTH) and PTH and DAPT-treated *Bmi-1^-/-^* mice (KO+PTH+PAPT) for determining the expression of Nortch1 and jagged1. The expression is calculated as a ratio to the GAPDH mRNA level and shown relative to the levels in vehicle-treated WT mice. For each genotype, n = 6; *: *p*<0.05, **: *p*<0.01, compared to vehicle-treated WT mice; #: *p*<0.05 compared to vehicle-treated *Bmi-1^-/-^* mice.

To gain insights into the underlying mechanisms, we investigated whether the improvement of haematopoietic defects occurred in Bmi1 deficient mice by PTH administration is associated with activated Notch signaling, which is crucial for haematopoietic stem cells [28]. Expression of the Notch ligand Jagged1 and the Notch intracellular domain (NICD) was thus examined by immunohistochemistry and Western blots. At 4 weeks of age, the number of Jagged1-positive cells, the percentage of Notch intracellular domain (NICD)-positive bone marrow cells and protein expression levels of Jagged1 and NICD in bone tissue were decreased significantly in vehicle-treated *Bmi1^-/-^* mice when compared to the wild-type counterparts (Figs. 6A–G). Administration of PTH1-34 increased the levels significantly in *Bmi1^-/-^* mice (Figs. 6A–G). To further demonstrate whether PTH administration activated Notch signaling, the 4-week-old *Bmi1* ^-/-^ mice were injected daily with PTH1-34 alone or with both PTH1-34 and DAPT, a Notch inhibitor, for 2 days, RNAs were isolated from mouse long bones, the expression of Notch1 and Jagged1 was examined at mRNA levels by real-time RT-PCR. The gene expression levels of Notch1 and Jagged1 were down-regulated in vehicle-treated *Bmi1^-/-^* mice and up-regulated in PTH-treated *Bmi1^-/-^* mice, however, the up-regulation of PTH on both Notch1 and Jagged1 was blocked by the DAPT administration. These results indicated that the Notch pathway is inhibited by *Bmi1* deletion, but activated by PTH administration.

## Discussion

It was previously shown that defects in haematopoiesis in *Bmi1*-null mice are due to severely impaired HSC self-renewal [7], but it is unclear whether the defects are associated with an impaired bone marrow microenvironment. It was reported that PTH not only exerts bone anabolic action by stimulating osteoblastic bone formation, but also promotes haematopoiesis by improving the bone marrow microenvironment [5], [16]–[18]. In the present study, we have investigated whether PTH administration rescues the haematopoietic defects caused by Bmi1 deficiency though improving the haematopoietic microenvironment. Our results demonstrate that administration of PTH1-34, a drug currently used for treating osteoporosis, partially rescues haematopoietic defects in *Bmi1*-deficient mice by improving the bone marrow microenvironment. These results imply that the haematopoietic defects caused by *Bmi1* deficiency are not only because of reduced HSC self-renewal, but also due to an impaired haematopoietic microenvironment.

Consistent with the previous findings [11], we found here that compared to the wild-type counterparts, *Bmi1*-deficient mice displayed i) defects in osteoblastic bone formation, including decreased trabecular bone volume, osteoblast number and ALP- or type I collagen-positive areas; ii) down-regulated ALP, osteocalcin and Runx2 expression in the bone; and iii) increased adipocytes and up-regulated PPARγ expression in the bone marrow. In addition, we found that *Bmi1* deficiency resulted in down-regulated expression of the PTHR and the IGF-1 in the bone. Our results also demonstrated that PTHR was localized in osteoblasts and stromal cells in bone marrow, but not in bone marrow haematopoietic cells. PTHR gene was localized in osteoblasts demonstrated by in situ hybridization [29]. Previous studies indicated that PTHR is a crucial mediator of bone-forming action of PTH as targeted expression of the constitutively active PTHR led to increased osteoblast function in trabecular bone and at the endosteal surface of cortical bone [30] and IGF-1 is required for the anabolic effect of PTH on bone formation as PTH had little effects on IGF-1-null mice [31]. Thus, the bone anabolic action of Bmi1 on osteoblastic bone formation is also associated with the regulation of PTHR and IGF-1.

Some of our findings appear to be slightly different from those in a previous report [7]. The previous study examined the alterations of bone marrow haematopoietic cells and peripheral blood cells in 2-month old *Bmi1* ^-/-^ and wild-type mice. The results showed that *Bmi1* ^-/-^ mice have significant less HSC frequency in the bone marrow and an average 10-fold less total HSCs when the total numbers of bone marrow cells were taken into account [7]. It was also found that in the peripheral blood, there were a normal number of myeloid cells but a smaller number of lymphocytes [7]. We examined 1-month old mice. The results showed that *Bmi1* ^-/-^ mice had insignificant fewer HSCs and HPCs in the bone marrow and 4-5-fold less total HSCs and HPCs when the total numbers of bone marrow cells were taken into account. Although the amount of granulocytes, red blood cells and platelets in the peripheral blood was not altered significantly, the numbers of lymphocytes and some other white blood cells were decreased dramatically in *Bmi1*-deficient mice (Fig. 5). The differences between this and the previous study could be due to that the severity of haematopoietic defects was lighter in 4-week-old Bmi1^-/-^ mice than 2-month-old Bmi1^-/-^ mice as previous report [7]. Related to this, we found that bone marrow cells were less, but adipocytes were more in 4-week old *Bmi1* ^-/-^ mice than 2-week-old ones [11]. These results indicate that Bmi1 deficiency results in progressive defects in haematopoiesis as the mice develop and age.

Importantly, we found that PTH1-34 administration partially reversed premature osteoporosis occurred in *Bmi1*-deficient mice. PTH1-34 administration increased trabecular bone volume, osteoblast number and activity, up-regulated ALP, osterix, osteocalcin, Runx2, PTHR and IGF1 expression in the bone, and reduced the number of adipocytes and PPARγ expression in the bone marrow. Runx2 is essential for the differentiation of osteoblasts from mesenchymal precursors [32]–[34]. Osterix, which acts downstream of Runx2, is a zinc-finger-containing transcription factor essential for embryonic osteoblast differentiation and bone formation [35]. PPARγ is a critical transcription factor involved in adipogenic differentiation [36]. As reported previously [11], our results showed that the Runx2 and osterix levels were downregulated, whereas the PPARγ level was upregulated in the bone tissues from *Bmi1* ^-/-^ mice. More importantly, PTH1-34 administration upregulated the Runx2 and osterix protein levels and downregulated the PPARγ levels in the bone tissues from *Bmi1* ^-/-^ mice. In osteoblasts, the binding of PTH to PTHR activates adenyl cyclase and phospholipase, leading to formation of cAMP and a subsequent increase in intracellular calcium concentration as well as activation of PKC, promoting osteoblastic bone formation [30]. Intermittent treatment with PTH induces an increase in IGF-1 expression in the bone tissues from both mice and rats [37], [38]. The anabolic effect of PTH depends on the expression of IGF-1 as PTH had no effect in IGF-1 null mice and was unable to induce important target genes for osteoblastic bone formation [31]. The current study not only demonstrated that PTHR and IGF-1 protein levels were downregulated in bone tissues from *Bmi1* ^-/-^ mice, but also showed that both proteins were upregulated in the bone tissues upon PTH1-34 administration. Consequently, the administration increased osteoblastic bone formation at least in part by stimulating osteoblast differentiation and inhibiting adipocyte differentiation through PTHR and IGF-1.

Studies using genetically altered animal models that could activate or destroy osteoblastic cells suggest that osteoblasts contribute to the HSC niche [5], [39], [40]. On the other hand, PTH mediated activation of osteoblasts resulted in a significant expansion of the HSC pool and led to the realization that targeting cells of the osteoblastic lineage is a potential therapeutic approach to enhance stem cell-based therapies [5], [16]–[18]. Base on that PTH1-34 administration increased osteoblastic bone formation in *Bmi1*-deficient mice, we assessed whether PTH1-34 administration could also rescue defects in haematopoiesis caused by Bmi1 deficiency. Our results revealed that the total numbers of bone marrow cells, the fraction of Sca-1^+^c-kit^+^Lin^+^ HPCs relative to the total number of bone marrow cells, and the numbers of white blood cells and granulocytes in peripheral blood all increased significantly after PTH1-34 administration. Although the fraction of Sca-1^+^c-kit^+^Lin^-^ HSCs relative to the total number of bone marrow cells did not increased significantly, total HSCs were increased 2.7-fold, and total HPCs increased 3-fold when the total numbers of bone marrow cells were taken into account. This reversal of haematopoietic defects was consistent with the increased osteoblast number and activity in PTH-treated *Bmi1* deficient mice. Furthermore, we also found that osteopontin-positive bone matrix area, the number of Jagged1-positive cells, percentage of NICD-positive bone marrow cells and protein expression levels of Jagged1 and NICD in bone tissue were decreased significantly in vehicle-treated *Bmi1* ^-/-^ mice, and all increased significantly in Bmi1^-/-^ mice by the administration of PTH1-34, whereas the up-regulation of PTH on both Notch1 and Jagged1 gene expression was blocked by the Notch inhibitor DAPT administration. Osteopontin is an important component of the HSC niche in which it participates in HSC location and acts as a physiologic-negative regulator of HSC proliferation [41]. In mice in which the PTHR was activated in osteoblastic cells only, osteoblastic cells were increased in number and produced high levels of Jagged1, the activated NICD was increased in the HSC fraction in vivo, and Notch inactivation by DAPT blocked HSC expansion in vitro [5]. Results from the present study indicate that PTH administration partially rescues haematopoietic defects in Bmi1 deficient mice by improving haematopoietic microenvironment and activating the Notch pathway.

In conclusion, this study shows that PTH administration increased osteoblastic bone formation and partially repaired the haematopoietic defects in *Bmi1*-deficient mice. The results indicate that haematopoietic defects caused by Bmi1 deficiency are not only because of reduced HSC self-renewal, but also because of impairment in the haematopoietic microenvironment. This study also reveals a potential value of PTH1-34, an anabolic drug for osteoporosis, for repairing haematopoietic deficiency.
